# Bilateral Clavicle Fractures - A Rare Injury

**DOI:** 10.7759/cureus.11449

**Published:** 2020-11-11

**Authors:** Kishore Vellingiri, Hariprasad Seenappa

**Affiliations:** 1 Department of Orthopaedics, Sri Devaraj Urs Medical College, Sri Devaraj Urs Academy of Higher Education and Research, Kolar, IND; 2 Department of Orthopaedics, Sri Devaraj Urs Medical College, Sri Devaraj Urs Academy of Higher Eduacation and Research, Kolar, IND

**Keywords:** bilateral clavicle fracture

## Abstract

Both surgical or nonsurgical management of bilateral clavicle fractures have advantages and disadvantages. Hence a patient must be advised for shared decision making. Our patient was a painter by occupation with a left-sided dominant hand. Considering his job and financial constraints, we fixed his left clavicle fracture surgically and treated his right-sided clavicle fracture conservatively. We suggest based on our report concerning this rare injury, is that not all fractures need to be fixed surgically. Patient needs and other factors should be taken into consideration before taking the patient for surgical management.

## Introduction

Bilateral clavicle fractures comprise about 0.43% of all clavicle fractures, with an incidence of 0.011-0.017% [1]. As it is a very rare injury, there is no general indication of treating this type of injury surgically or non- surgically. The patient in this case report was diagnosed with a closed bilateral clavicle fracture in a single setting. But here, we concentrated on patient variables rather than surgically repairing the fracture. Since surgical or non - surgical management has advantages and disadvantages, the patient must be counseled for achieving shared decision making.

This case report was presented as a poster presentation, and the abstract of this article is published in the online supplement of the conference journal (poster presentation: Vellingiri K, Seenappa H. Bilateral clavicle fractures - a rare injury. 33^rd^ Bangladesh Orthopaedics Society, BOSCON 2020 at 3-5 Feb, Dhaka, Bangladesh). 

## Case presentation

**Figure 1:**
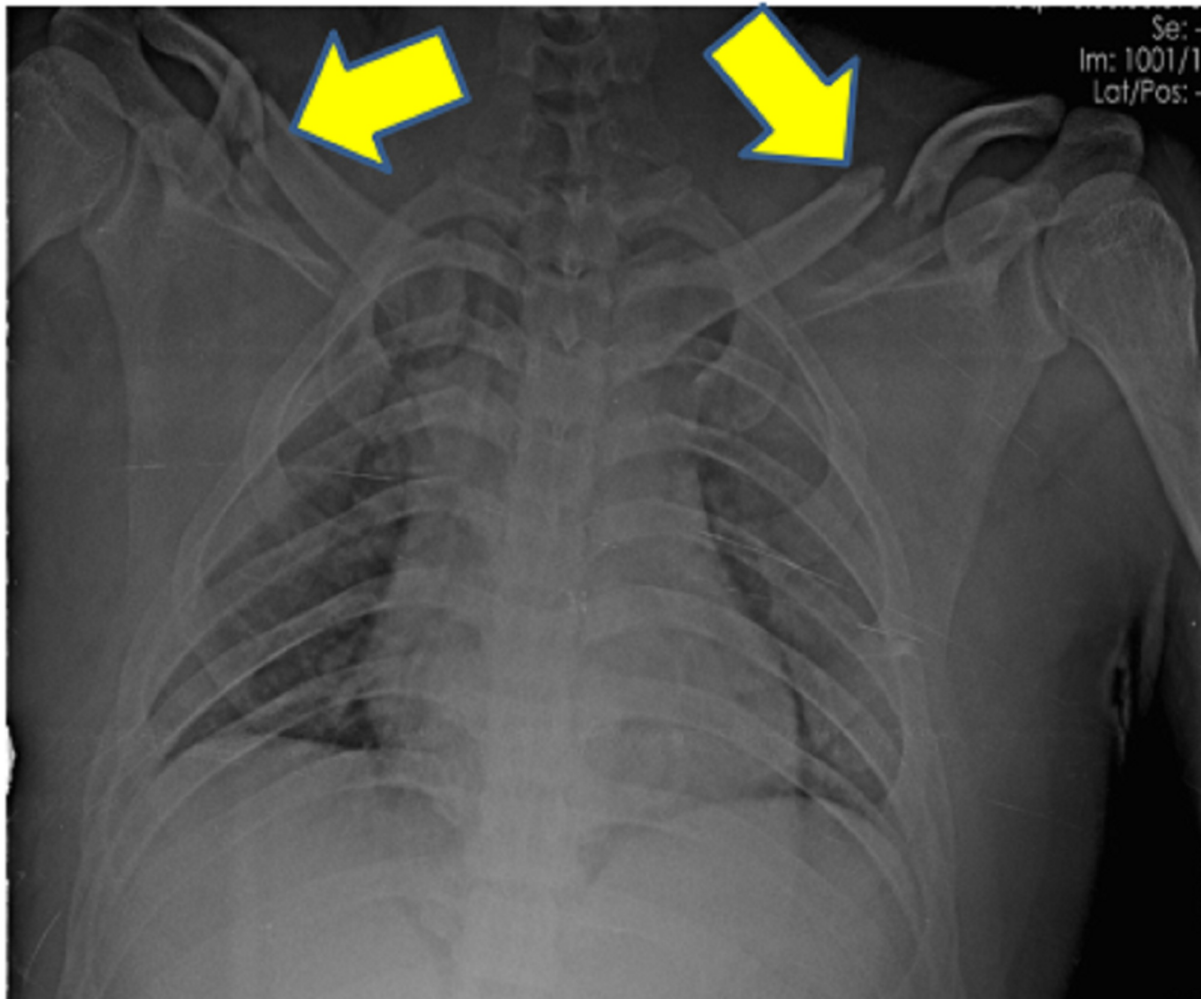
Plain radiographs of the chest with bilateral shoulder anteroposterior view showing bilateral clavicle fracture

A 22-year-old left-handed male patient, a painter by occupation, presented to RL Jalappa Hospital and Research Centre affiliated with Sri Devaraj Urs Medical College, Tamaka, Kolar, Karnataka, India. The patient gave the alleged history of the road traffic accident while traveling in a four-wheeler, which toppled and caused the patient sustained an injury to both his shoulders. On examination swelling, crepitus, and tenderness were present over the junction of the middle and lateral third junction of the right and left clavicle. The range of motion of both shoulder joints was painful and restricted. No distal neurovascular deficits were found. The patient was clinico-radiologically diagnosed as having closed bilateral clavicle fractures without neurovascular deficits, as shown in Figure 1. The patient and his relatives** **got an explanation about the severity of the injury, associated complications, and the need for surgery in their own understandable language. The patient was kept on the shoulder arm pouch on both sides with ice pack application and started on analgesics, anti-edema measures like bromelain, trypsin, and rutoside tablets. The patient and his relatives were asked about their demographic details, and surgical options were explained to them by a team of orthopedicians. The rehabilitation protocol, complications associated with both conservative versus non-conservative management were explained. After obtaining consent, the patient had surgery on the open reduction and internal fixation with a locking compression plate for the left side clavicle. The patient was not willing to get surgery for the right clavicle due to his financial constraints. The right clavicle fracture was treated conservatively by using a shoulder arm pouch application for three weeks. Range of right-shoulder activity limited to two weeks of pendulum exercise and active movements up in the horizontal plane during the first six weeks.

**Figure 2:**
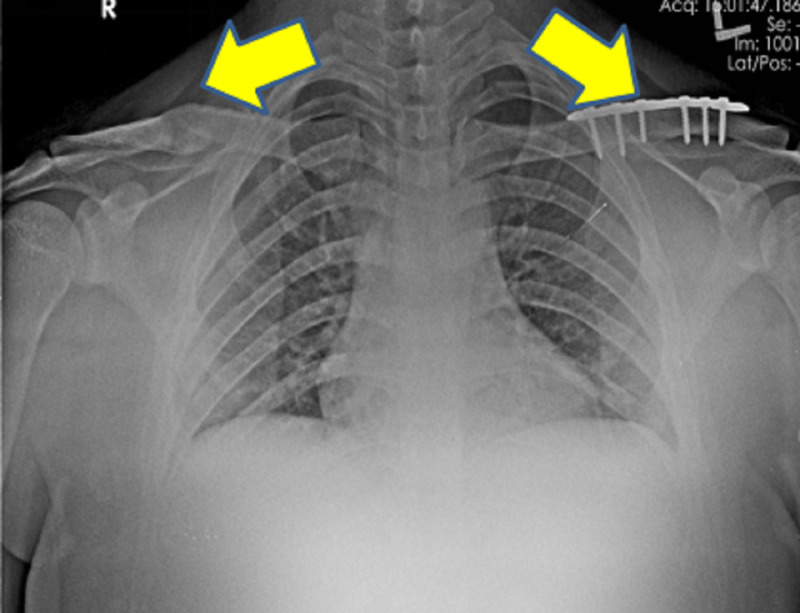
The plain radiograph of the chest with bilateral shoulder anteroposterior view at six months follow up showing locking compression plate in position for left clavicle fracture and united right clavicle fracture

**Figure 3:**
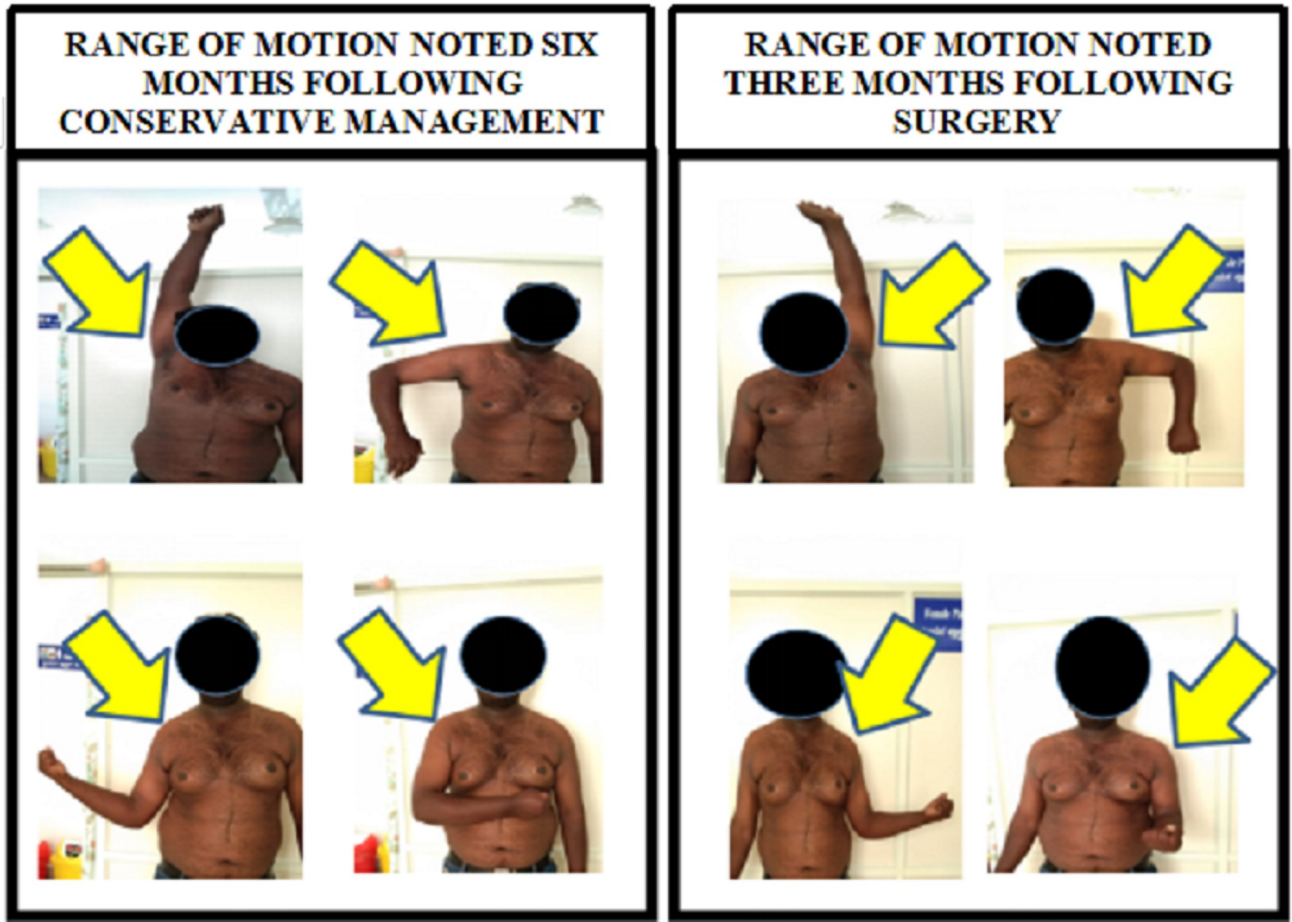
Clinical picture comparing the range of motion over operative (left) vs. non-operative (right) side

Post-operation of the left clavicle, the patient was able to raise the right arm above his shoulder. The patient was started on analgesics, anti-edema medications, and intravenous administration of augmentin 1.2 g twice daily for two days and started prophylactically oral amoxiclav 625 mg twice a day for seven days to prevent surgical site infection. Active ranges of motion were full with reduced pain. During one month follow-up, the radiograph over the left side showed better uniting of the fracture than the right side. The patient was allowed to return to work to his normal activity of painting three months post-operation. Figure 2 demonstrates the plain radiograph of the chest with bilateral shoulder anteroposterior view at six months follow up. Clinical picture comparing the range of motion over operative (left) versus non-operative (right) side shown in Figure 3. He was discharged from the follow up at six months.

## Discussion

Clavicle fractures incidents worldwide vary from 24 fractures per 100,000 population per year to 71 per 100,000, showing the increasing trend [2]. These injuries occur more commonly in young adults and children, mostly in men younger than 25 years [2]. Bilateral clavicle fractures could be missed because of their association with more severe chest injuries or a more symptomatically displaced fracture on one side or due to inadequate chest radiographs. Lakhotia, et al. reported in their case series that fractures with various energy mechanisms were treated conservatively and returned to good functional outcomes without any complications [3]. Kulkarni, et al. demonstrated in their study that intramedullary K-wire fixation at a single setting on both clavicle fracture showed good bridging callus formation and the full range of motion without any disturbance of daily activities [4]. Bajuri, et al. proposed using a 3.5 mm reconstruction plate to clavicle fracture bilaterally, and they noted good union and no limitation in range of motion and early returning to daily activities [5]. Qi, et al. study demonstrated how the right clavicle fixation was done with a reconstruction locking plate and a hook plate, and the left clavicle was fixed with an S-shaped locking plate. They achieved bone union at three months following surgery and concluded that double-plate fixation successfully achieved excellent long-term outcomes [6]. According to various authors, surgical choice differs; in our study, we emphasize considering patient factors before employing surgical management. A retrospective comparative study hypothesized that functional outcomes do not differ in both surgically versus non-operatively treated group. No differences in complications or poor outcomes were noted in surgical versus non-operative treatment [7].

Social factors proved to be greater predictors of outcome rather than other patient attributes or injury features. Management of clavicle fractures should be done after assessing patient expectations and activity levels [7]. Burnham, et al., in their study stressed the importance of reviewing anatomy, a system of classification and injury mechanisms for mid-shaft clavicle fractures, and comparing various treatment options. A systematic review based on the Cochrane database proposed that treatment options must be chosen on an individual patient basis, after carefully considering the benefits and hazards of each intervention and patient preferences. There is low-quality evidence that surgical treatment has no additional benefits in terms of function, pain, and quality of life compared to conservative treatment [8]. There is a need to consider the balance of risks between individual outcomes like wound infection, dehiscence, or hardware irritation in operated patients as well as symptomatic malunion, shoulder stiffness in conservatively management patients [9]. As Ahmed, et al. highlighted, there remains inconsistent evidence regarding the best treatment for managing displaced mid-shaft clavicle fractures [10]. Our patient was a left-handed painter by occupation, and considering his job and financial constraints, we fixed his left clavicle fracture surgically and treated his right-sided clavicle fracture conservatively.

## Conclusions

Successfully treating bilateral clavicle fractures using two different modalities of the same patient is a challenge. Our observation based on our report of this rare injury is that not all fractures need to be fixed surgically. We need to analyze patient needs and other factors before taking the patient for surgical management. The patient in our report had an excellent outcome in rehabilitation in the early period and returned to function following surgical intervention.
